# Improved *In Vitro* Culture of *Plasmodium falciparum* Permits Establishment of Clinical Isolates with Preserved Multiplication, Invasion and Rosetting Phenotypes

**DOI:** 10.1371/journal.pone.0069781

**Published:** 2013-07-22

**Authors:** Ulf Ribacke, Kirsten Moll, Letusa Albrecht, Hodan Ahmed Ismail, Johan Normark, Emilie Flaberg, Laszlo Szekely, Kjell Hultenby, Kristina E. M. Persson, Thomas G. Egwang, Mats Wahlgren

**Affiliations:** 1 Department of Microbiology, Tumor and Cell Biology, Karolinska Institutet, Stockholm, Sweden; 2 Department of Laboratory Medicine, Karolinska Institutet, Huddinge, Sweden; 3 Med Biotech Laboratories, Kampala, Uganda; Bernhard Nocht Institute for Tropical Medicine, Germany

## Abstract

To be able to robustly propagate *P. falciparum* at optimal conditions *in vitro* is of fundamental importance for genotypic and phenotypic studies of both established and fresh clinical isolates. Cryo-preserved *P. falciparum* isolates from Ugandan children with severe or uncomplicated malaria were investigated for parasite phenotypes under different *in vitro* growth conditions or studied directly from the peripheral blood. The parasite cultures showed a minimal loss of parasite-mass and preserved percentage of multiple infected pRBCs to that in peripheral blood, maintained adhesive phenotypes and good outgrowth and multiplication rates when grown in suspension and supplemented with gas. In contrast, abnormal and greatly fluctuating levels of multiple infections were observed during static growth conditions and outgrowth and multiplication rates were inferior. Serum, as compared to Albumax, was found necessary for optimal presentation of PfEMP1 at the pRBC surface and/or for binding of serum proteins (immunoglobulins). Optimal *in vitro* growth conditions of *P. falciparum* therefore include orbital shaking (50 rev/min), human serum (10%) and a fixed gas composition (5% O_2_, 5% CO_2_, 90% N_2_). We subsequently established 100% of 76 frozen patient isolates and found rosetting with schizont pRBCs in every isolate (>26% schizont rosetting rate). Rosetting during schizogony was often followed by invasion of the bound RBC as seen by regular and time-lapse microscopy as well as transmission electron microscopy. The peripheral parasitemia, the level of rosetting and the rate of multiplication correlated positively to one another for individual isolates. Rosetting was also more frequent with trophozoite and schizont pRBCs of children with severe versus uncomplicated malaria (p<0.002; p<0.004). The associations suggest that rosetting enhances the ability of the parasite to multiply within the human host.

## Introduction

Cytoadherence and rosetting in *Plasmodium falciparum* malaria, the binding of parasitized erythrocytes (pRBC) to endothelial cells and uninfected erythrocytes (RBC), may cause excessive micro-vascular sequestration, obstruction of blood flow and severe disease [1], [2], [3], [4], [5]. Malaria is a selective force on human populations and erythrocyte polymorphisms have evolved that provide resistance to severe malaria through different mechanisms [6], [7], [8], [9], [10], [11], [12]. Rosettes are smaller and weaker in blood group O erythrocytes compared with groups A, B, and AB [13], and blood group O has been found to confer resistance to severe malaria due to reduced rosetting [14], [15]. Similarly, CR1 deficient, thalassemic and hemoglobin S- or C-containing RBCs possess an impaired ability to adhere to pRBCs and are thought to account for a protective role in malaria [16]. The decreased rosetting is associated with the lack of CR1, the small size of the thalassemic RBCs and with distortion of the mechanical properties and altered PfEMP1 display of HbS-containing RBCs [12], [17]. However the biological advantage of the rosetting phenomenon for the parasite has yet not been established.

Both the peripheral parasitemia and the sequestered biomass of *P. falciparum* are higher in patients with severe malaria than in patients with uncomplicated disease [18]. This could be accounted for by differences in host immune responses or in the expression of erythrocyte receptors, but underlying variations in the ability of the parasite to multiply could equally well lead to differences in parasite-mass. A correlation between parasite multiplication rates and severe malaria has previously been reported [19] and a relationship between rosette formation and parasite density has been observed in *Saimiri* monkeys [20] but the underlying mechanisms are not known. Discrepancies in growth may, amongst others, rely on the rates of replication, the number of formed merozoites and the invasion ligand repertoire presented by the merozoites. Rosettes formed by schizont-containing pRBCs have previously been found with the primate malaria parasite *P. fragile* [21] and *P. falciparum* [22]. In children with severe disease the parasites have been found to form rosettes and giant rosettes at a high frequency and bind to multiple receptors [1], [23], [24], even though contradictory results have been reported [25], [26], making this biological phenomenon attractive to investigate for its potential role in generating high levels of parasitemia. Indicative of a role of rosetting in merozoite invasion is also the occasional observation of several newly infected RBCs surrounding a bursting schizont and the presence of ring-infected RBCs in the microvasculature of children who succumb in cerebral malaria [5].

Measures of rosetting, multiplication and RBC invasion have traditionally been hampered by difficulties adapting fresh and cryo-preserved clinical isolates to *in vitro* culture. Loss of parasite mass due to poor outgrowth and low multiplication rates can have devastating effects on both genotype and phenotype representations within heterogeneous samples. Similarly, phenotypes such as rosetting and cytoadhesion may be lost due to lack of specific host factors or limitations in mimicking the rheological and micro-aerophilic environment of the vasculature. *P. falciparum* propagated *in vitro* under static conditions employing the classical candle jar method looses its capacity to form knobs at the surface of the pRBC, adhere to host cells and also to switch PfEMP1 expression [27], [28], [29], [30]. In previous work it has been suggested that serum-lipid components are required for the final relocation of the PfEMP1 molecule from the Maurer’s clefts to the pRBC surface [31], [32]. Other factors such as complement factor D, albumin and naturally occurring antibodies to the anion transport protein band 3 have been attributed a role in rosetting [33]. In addition, the necessity of the presence of human serum for the formation of rosettes has been shown for both laboratory strains as well as patient isolates, where rosettes frequently contain non-immune immunoglobulins [1], [4], [34], [35], [36], [37], [38]. To be able to robustly investigate *P. falciparum* it is therefore needed to propagate the parasite at conditions *in vitro* that mimic the environment of the human microvasculature as much as possible.

Optimized growth conditions as established herein allowed us to achieve an outgrowth of 100% of 76 patient isolates and ensured correct display of the parasite derived molecules on the pRBC surface. In order to analyze a possible correlation between rosetting and multiplication rate, parameters such as multiplication, outgrowth, multiple infection and rosetting rates with trophozoite as well as schizont stages for isolates derived from severe malaria patients were compared to those of uncomplicated cases. We demonstrate that rosetting during schizogony, with a concordant invasion of uninfected RBCs upon rupture, is a phenomenon, which is observed in clinical isolates but is less common in laboratory clones. Our data imply, that the ability of the parasite to multiply might be enhanced by rosetting within the malaria patient, suggesting the close proximity to uninfected RBCs in rosettes might facilitate merozoite invasion in *P. falciparum* malaria.

## Results

### Advantageous *in vitro* Growth Conditions for Clinical *P. falciparum* Isolates

**Figure 1:**
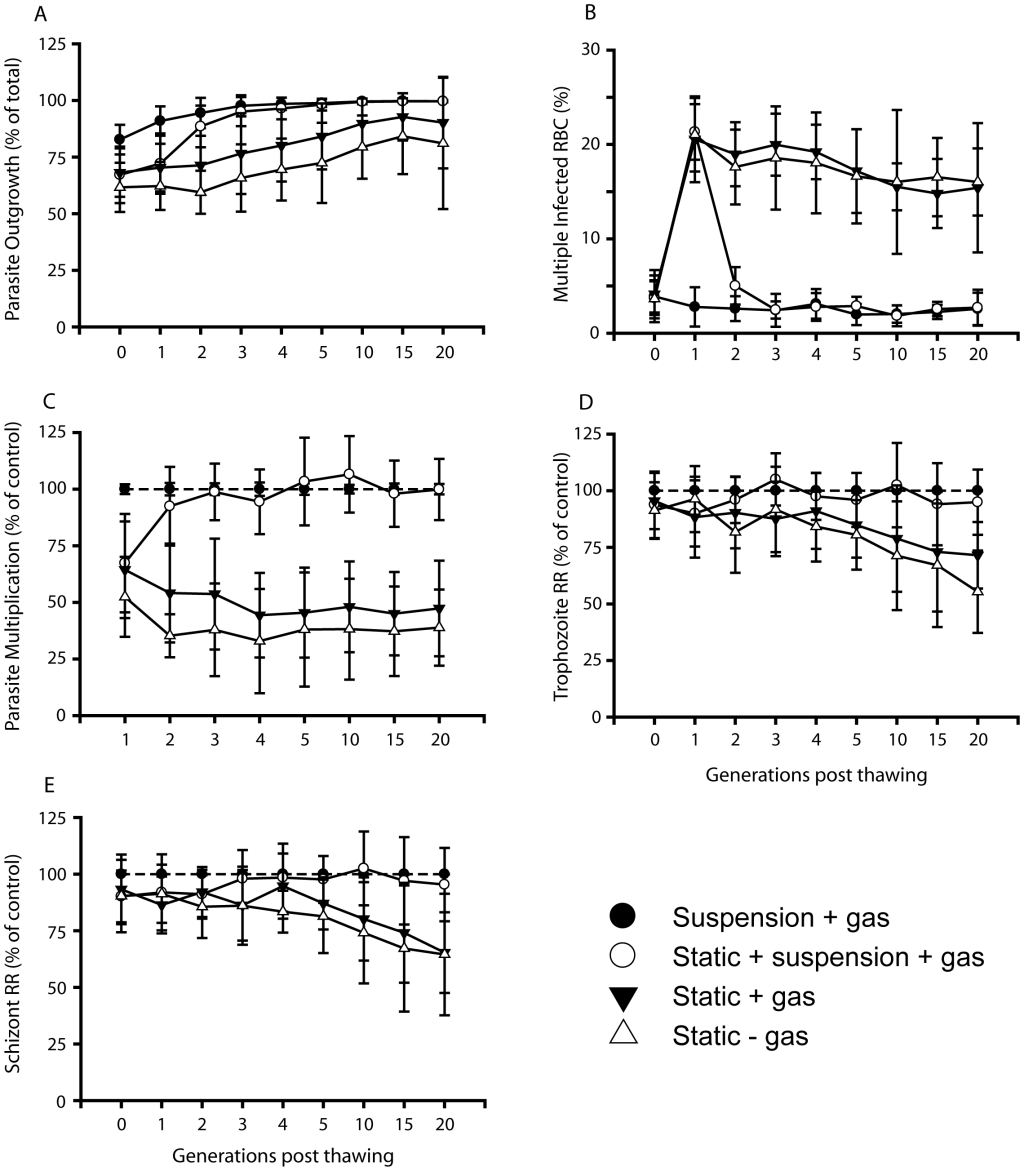
Parameters of patient isolates grown under different conditions. Improved parasite outgrowth and multiplication, lower frequency of multiple infected erythrocytes and preserved rosetting phenotype in isolates grown in suspension with fixed gas composition. Ten isolates, all subjected to four different *in vitro* growth conditions for twenty parasite generations, were scored for percentage viable (versus pyknotic) parasites (outgrowth) (A), rates of multiple infected RBCs (B), parasite multiplication rates (PMR) (C), trophozoite rosetting rates (TRR) (D) and schizont rosetting rates (SRR) (E). The different conditions were 1) growth in suspension (50 rev/min) with fixed gas composition (5% O2 and 5% CO2 in N2) (•), 2) the same but with first generation grown under static conditions (○), 3) static growth with fixed gas (▾) and 4) static growth with candle jar technique (Δ). Data are presented as mean values of the ten isolates and error bars represent standard deviations. For full multiple pairwise comparisons of the four culturing conditions for each generation, please see Figure S1.

Optimal growth conditions are of importance when comparing the multiplication of parasites obtained from patients. A systematic evaluation was therefore undertaken to ensure that 100%, all of 76 cryopreserved clinical isolates (Table S1, Figure S1), endured during *in vitro* growth with minimal loss of parasite mass and with the preservation of the original parasite phenotype. A comparison of four different *in vitro* growth conditions was carried out based on 10 of the 76 isolates. Cultivation involving growth in suspension on an orbital shaker (50 rev/min) with the use of a fixed gas composition (5% O_2_, 5% CO_2_ and 90% N_2_) to maintain a stable micro-aerophilic environment was found preferred as compared to static cultivation with or without the addition of the gas mixture (candle-jar method). All isolates could thereby be established with a minimal loss of parasites during outgrowth (<20% loss, Figure 1A), with a reduction of multiple invaded erythrocytes to the levels observed in the peripheral blood of the patients, from ∼20% when grown statically to ∼3% when grown in suspension (Figure 1B), with high rates of parasite multiplication (Figure 1C) and a preserved capacity to form rosettes over time (Figure 1D, E) as compared to static conditions in the absence (two isolates failed to be adapted to *in vitro* growth under this condition) or presence of gas.

### Advantageous *in vitro* Growth Conditions for Formation of Rosettes in *P. falciparum* Strains

**Figure 2:**
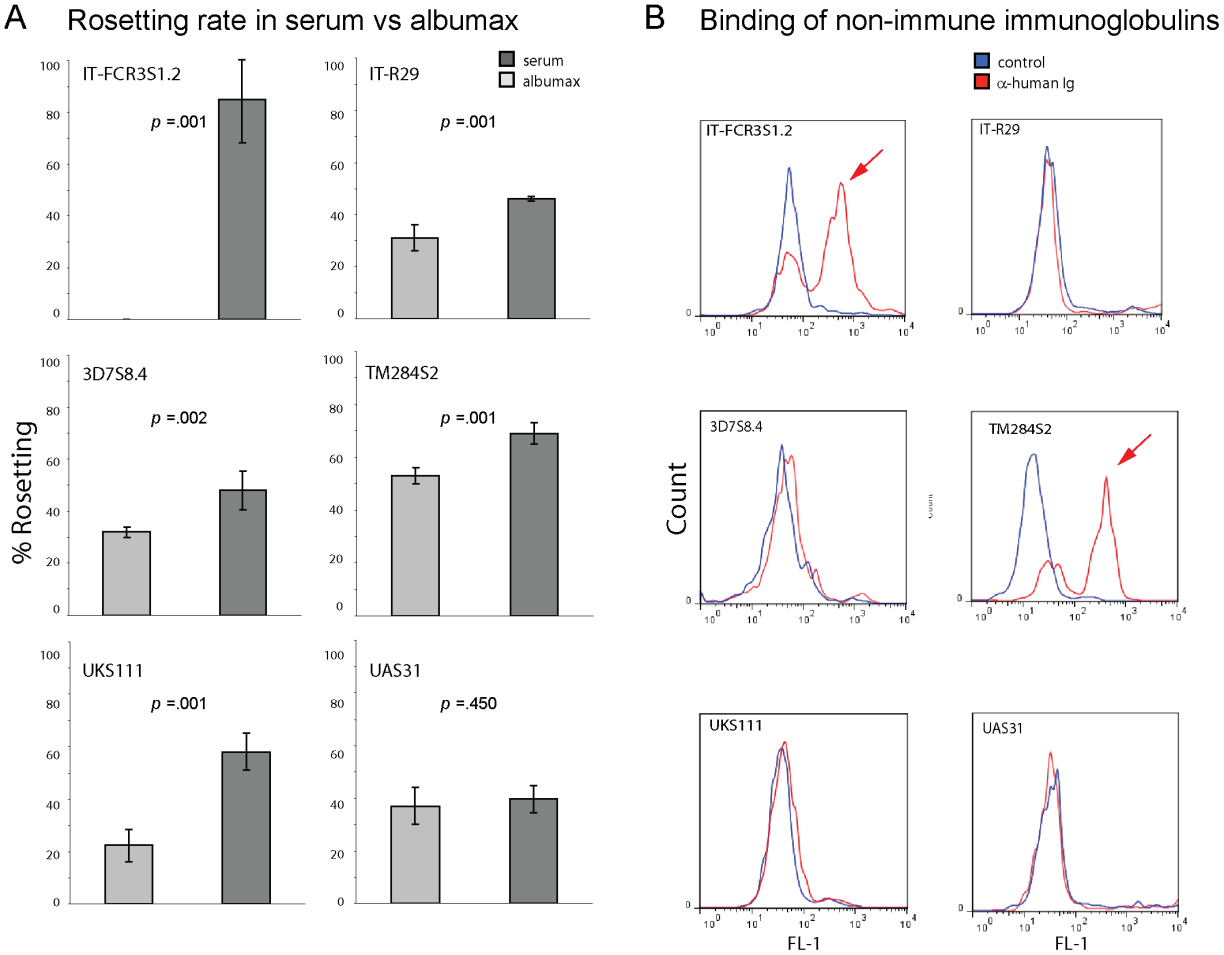
Rosette formation and binding of non-immune immunoglobulins in laboratory stains and patient isolates. A: Different parasite strains and patient isolates were grown in medium complemented with human serum or albumax. The rosetting rate at 34–38 h p.i. was assessed in three consecutive cycles in the different conditions (dark grey: presence of human serum, light grey: absence of human serum). Graph shows mean of three experiments with range. B: Binding of non-immune immunoglobulins in the corresponding parasite lines to the surface of the pRBC surface as visualized with an anti-human Ig antibody (red: anti-human Ig-Alexa, blue: control antibody), immunoglobulin binding pRBCs are marked with red arrows.

Growth conditions *in vitro* that resemble conditions met by the parasite *in vivo* are important for the correct development of the adhesive phenotype of the parasite. Different laboratory parasite strains (FCR3S1.2, R29, 3D7S8.4, TM284S2) and two patient isolates with a rosetting phenotype were propagated and analyzed in presence or absence of human serum. Parasites were adapted for three generations to medium with or without serum and afterwards monitored for their capacity to form rosettes during three consecutive generations. The dependence of rosetting on the presence or absence of human serum varied in different parasite strains (Figure 2A). FCR3S1.2 pRBCs did not show any rosettes when grown in absence of human serum, while the rosetting rate was around 80% in the presence of human serum. Switching of culture conditions rapidly re-established the rosetting phenotype in the presence of human serum, while it was lost by a change to conditions without human serum (data not shown). Rosetting disappeared partly in the parasite strain R29, 3D7S8.4, TM284S2 and the patient isolate UKS111 when no human serum was present. In contrast the patient isolate UAS31 did not show any loss in the ability to form rosettes when depleted of human serum. No correlation between the capacity to bind non-immune Ig to the pRBC surface and dependence of rosetting on presence of human serum was observed, except for the parasite strain FCR3S1.2 with a high capacity to bind non-immune human serum which completely lost the rosetting phenotype when human serum was removed from the cultures (Figure 2B).

### Importance of *in vitro* Growth Conditions for PfEMP1 Surface Expression by *P. falciparum* Strains

**Figure 3:**
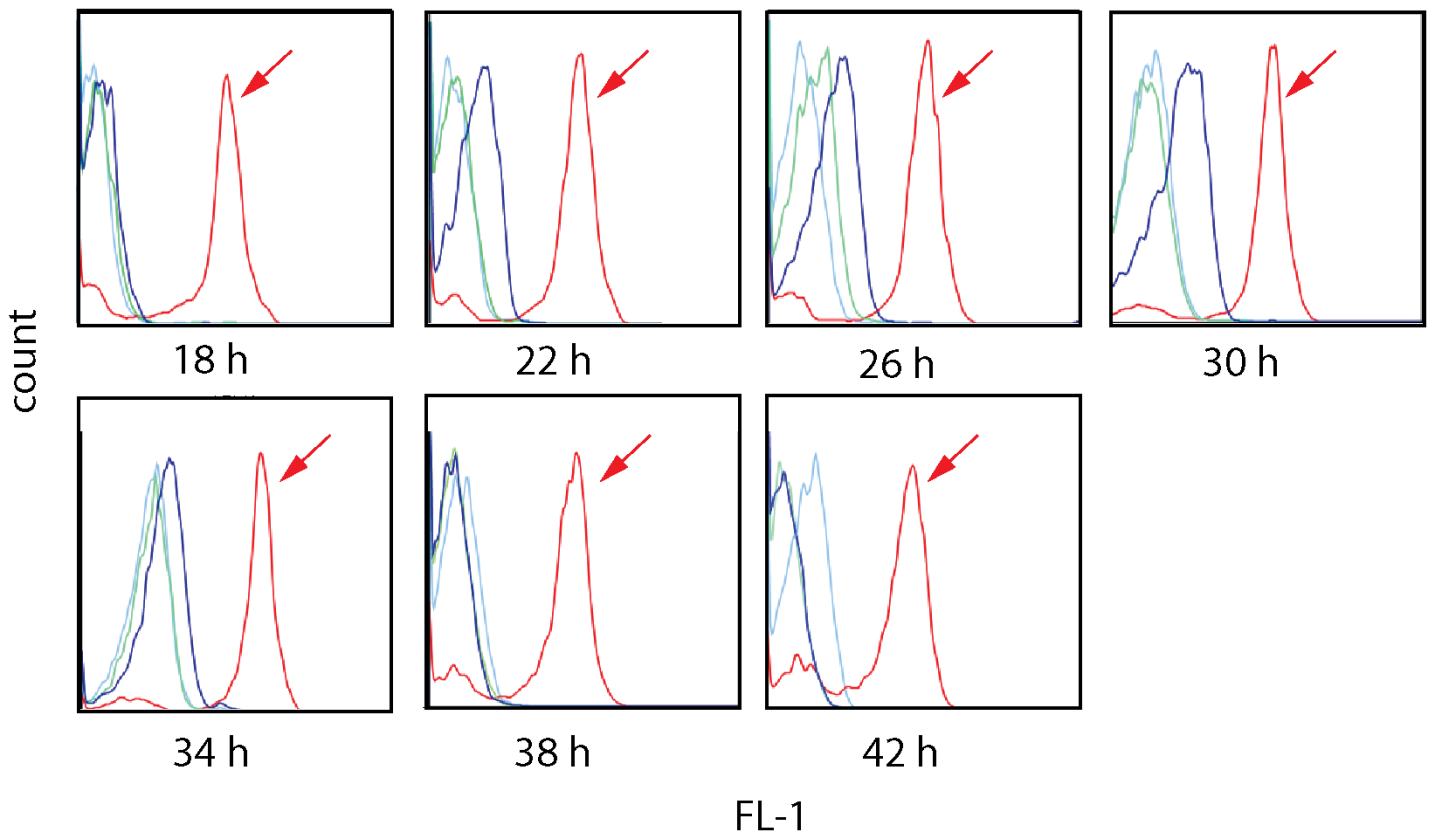
Surface expression of PfEMP1 in FCR3S1.2 in the presence or absence of human serum. FCR3S1.2 pRBCs were monitored with specific anti-PfEMP1 antibodies for surface expression of PfEMP1 as compared when grown in the presence of human serum (red,+arrow) or albumax (blue) at 7 different time points. Antibodies against a non-related control protein were used as controls (pRBCs grown in serum: light green; in albumax: light blue).

Expression of the parasite derived surface protein PfEMP1 was analyzed in the different growing conditions: The parasite strain FCR3S1.2 expressing the PfEMP1 encoded by the IT4var60 gene was grown in presence or absence of human serum and the exposure of PfEMP1 on the pRBC surface was monitored with a specific monoclonal antibody towards the DBL1α-domain of PfEMP1-IT4var60 [39] at 7 different time-points during the parasite’s life cycle (18, 22, 26, 30, 34, 38, 42 h pi.). Expression of PfEMP1 in FCR3S1.2 was significantly lower in pRBCs grown in the presence of Albumax as compared to human serum. PRBCs in Albumax had a decreased amount of PfEMP1 present on the surface. Moreover, PfEMP1 was expressed during a shorter time of the parasite’s life cycle, with expression only detected between 22 h and 34 h pi while pRBCs grown with human serum presented PfEMP1 between 18 h and 42 h pi (Figure 3).

### Clinical Isolates Rosette during Schizogony and Invade Bound Erythrocytes

**Figure 4:**
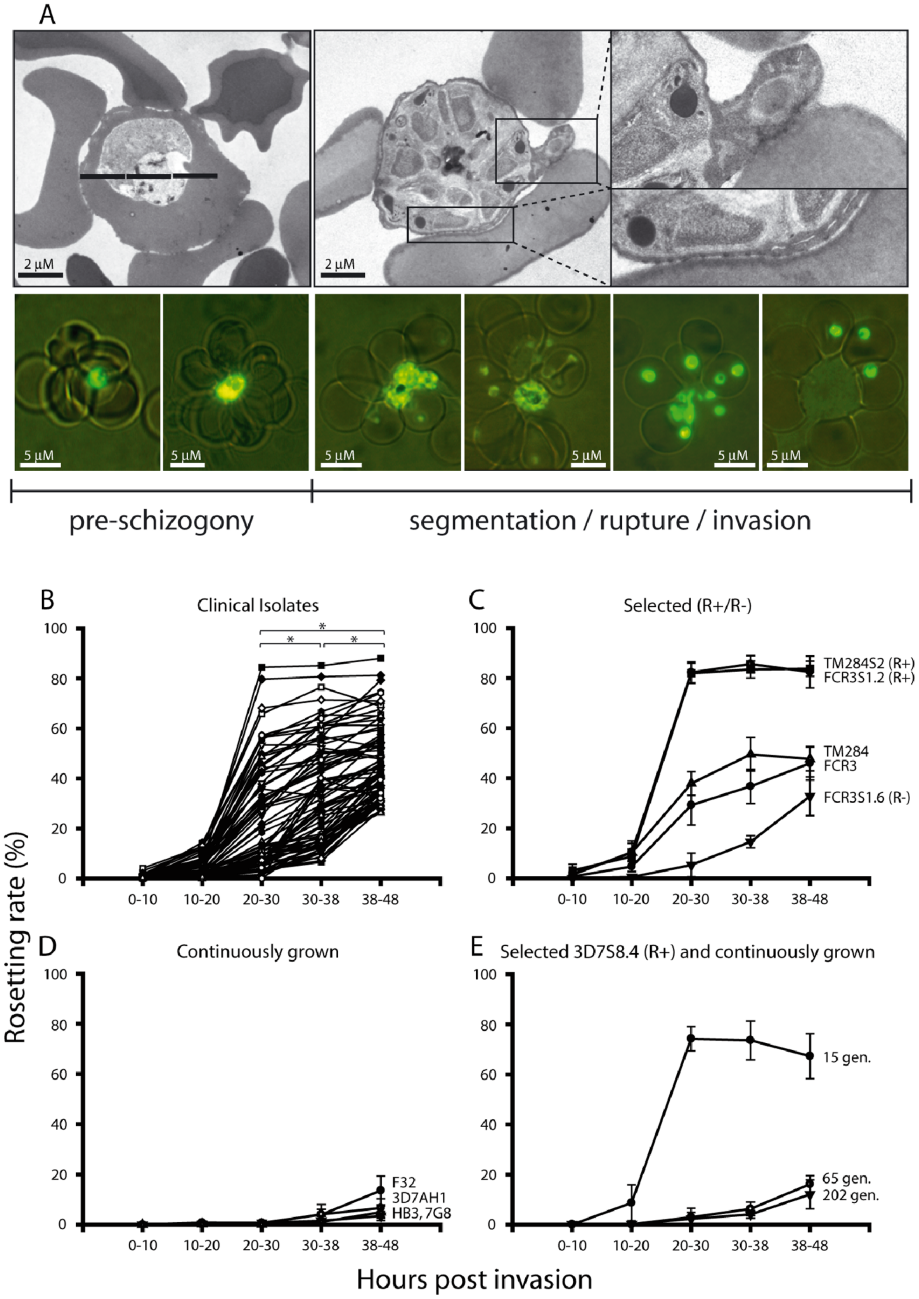
Rosette formation in clinical isolates and laboratory strains of *Plasmodium falciparum*. (A) Transmission electron micrographs of rosette forming trophozoite stage infected erythrocytes and the frequently observed rosetting at the time of schizogony in the clinical isolate UAS29. Live cell fluorescence during the course of rosetting in the isolate UAS31 showing rosetting during schizogony with a concordant invasion of bound uninfected erythrocytes upon rupture (parasites stained using acridine-orange and fluorescing in green, imaged using a Nikon Optiphot 2 Phase Fluorescence Microscope and a Hamamatsu Color Chilled 3CCD Camera at 100 x magnification). (B) Timing of rosette formation in 76 clinical isolates expressed as number of parasitized erythrocytes binding ≥2 uninfected erythrocytes. Each rosetting rate is the mean of the rosetting found during the three first consecutive generations post thawing. Stars indicate statistically significant higher levels of rosetting rate at schizont stage (p<0.05) compared to trophozoite stage. (C-E) Timing of rosette formation in laboratory clones and strains. Long-term propagated parasite strains not selected for rosetting or cytoadhesion were HB3, F32, 3D7AH1, FCR3 and TM284. Parasite clones selected for high or low rosetting (R+/R−) by micromanipulation were FCR3S1.2 (R+), FCR3S1.6 (R−), 3D7S8.4 (R+) and TM284S2 (R+). Rosetting rates are expressed as means with error bars representing the range of the biological replicates.

Investigating the timing of rosette formation in the 76 clinical isolates from Apac (Uganda) [40], [41] revealed that all isolates did form rosettes at time of schizogony (Figure 4B). The levels of schizont rosetting varied between individual isolates with a range of 26.2 to 88.0% (average of the first three consecutive parasite generations post thawing). A concordant invasion of bound uninfected red blood cells upon rupture was frequently observed with the fresh clinical isolates both by TEM and fluorescence microscopy (Figure 4A), as well as time-lapse capture employing a Nipkow spinning disc confocal microscope (Figure S2, Movie S1). The levels of schizont rosettes were thus higher than mid-trophozoite rosettes (20–30 h), the stage during which rosetting has previously routinely been monitored (Figure 4B).

### Permanence of the Trophozoite and Schizont Rosetting Phenotypes

Laboratory strains, some which had previously been selected for the trophozoite rosetting phenotype (TM284S2, FCR3S1.2), with high expression of PfEMP1 on the pRBC surface [24], were also shown to rosette to high degrees during schizogony (Figure 4C). Long-term propagated, non-selected parasite clones or lines (FCR3, TM284, HB3, 3D7AH1, 7G8, F32) or a parasite cloned for the lack of trophozoite-rosetting (FCR3S1.6) with little or no PfEMP1 on the surface of the pRBC, did however rosette, but at relatively low levels (Figure 4C–D). Further in one parasite cloned by micro-manipulation for rosetting (3D7S8.4), which was left to grow in the absence of enrichment, rosetting was lost after >60 generations (Figure 4E) concurring with the loss of PfEMP1 on the pRBC surface [42]. Taken together, parasites form rosettes to higher levels during schizogony than at trophozoite stage. This indicates that the levels of rosetting diminishes in the absence of adhesion-selection, immunological stimuli and/or loss of knob structures due to spontaneous deletion of the subtelomere on chromosome 2 [28].

### Rate of Parasite Multiplication Correlates Positively to the Rosetting Phenotype in Clinical Isolates

**Figure 5:**
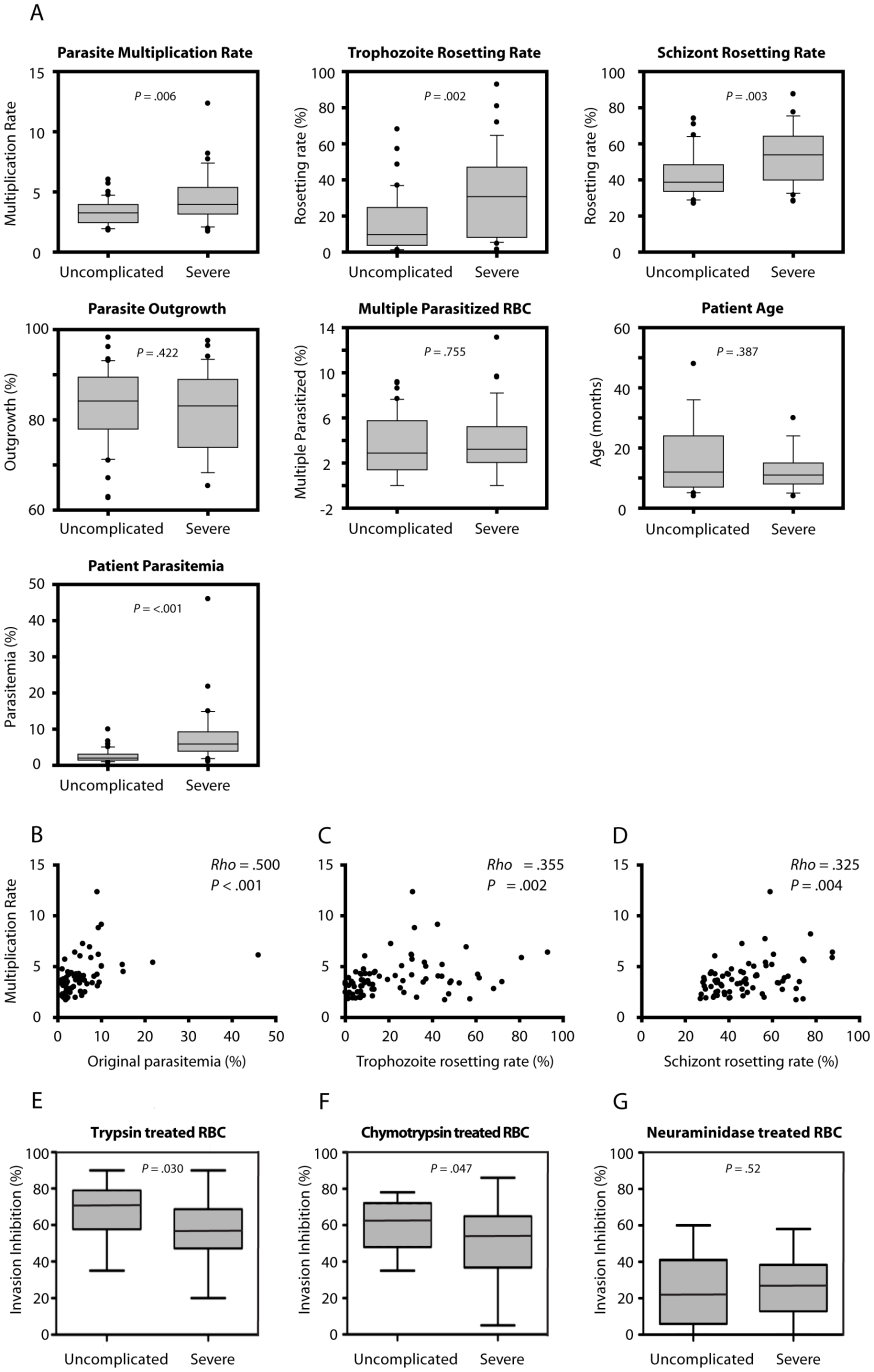
Phenotypic characteristics of clinical isolates and age of children with severe or uncomplicated malaria. (A) Boxed plots showing group comparisons of isolates originating from patients with severe (n = 36) and uncomplicated (n = 40) malaria in respect to patient age, parasitemia, growth and rosetting during the first generation post thawing. (B-D) Scatter plots showing Spearman rank correlations between parasite multiplication rates and original peripheral parasitemia of the patient or rosetting rates with mid-trophozoite or schizont infected RBCs for individual isolates. (E-G) Inhibition of invasion into trypsin-, chymotrypsin or neuraminidase treated erythrocytes by 11 *P. falciparum* isolates from children with uncomplicated malaria and 10 isolates of children with severe malaria.

Of the 76 clinical isolates, 36 originated from children with severe malaria and 40 from children with uncomplicated disease (Table S1). The severe cases suffered from respiratory distress, anemia and cerebral malaria and had overall higher levels of peripheral parasitemia than the uncomplicated group (mean ±95% C.I. 7.8% ±2.6% vs. 2.6% ±0.6% respectively). The fresh isolates from all of the patients were carefully monitored for several parasite features *in vitro*. Parameters such as outgrowth and the number of multiple parasitized RBCs did not vary between the two groups of children (Figure 5A), suggesting parasite RBC invasion selectivity as a poor determinant of severe disease. Significant differences were however found for the parasite multiplication rate in fresh erythrocytes as well as for rosetting, both at the stage of mid-trophozoites and schizonts (Figure 5A). Interestingly, positive and significant correlations of the multiplication rates of individual isolates to the original peripheral parasitemia at the time of blood collection were also observed (Figure 5B). Similarly, the ability to multiply correlated to the rosetting rates of both trophozoite and schizont-infected RBCs (Figure 5C–D).

### Different Invasion Pathways between Isolates

To further characterize the investigated isolates, 21 (11 uncomplicated, 10 severe) of the 76 clinical isolates were studied for their RBC receptor specificities in merozoite invasion inhibition assays. The RBCs were treated with trypsin, chymotrypsin or neuraminidase prior to the merozoite invasion was investigated. The majority of isolates used neuraminidase resistant pathways, and further also used trypsin and chymotrypsin sensitive pathways. Specificity of invasion was shown to differ slightly between isolates of children with severe or uncomplicated malaria in sensitivity to trypsin and chymotrypsin; *p* = 0.030 and *p* = 0.047, respectively (Figure 5E, F), while no difference in sensitivity towards neuraminidase treatment of erythrocytes (*p* = 0.52*)* was observed between the groups (Figure 5G).

### Parasites with a Rosetting Phenotype do not have a Growth Advantage *in vitro*

To analyze the correlation between rosetting and multiplication observed in the clinical isolates, laboratory isolates were grown in conditions favoring as compared to preventing rosetting. FCR3S1.2 pRBCs were cultured in the presence of anti-NTS-DBL1α_var2_ antibodies (goat purified IgG, mouse mAb) completely inhibiting the formation of rosettes. Growth rates were compared to the identical parasite grown in the presence of control antibodies. All cultures were grown parallel static and shaking. For the laboratory strain FCR3S1.2 no growth advantage and no correlation between rosetting and multiplication could be observed (Figure S3).

## Discussion

Rosetting of *P. falciparum* in the microvasculature has been established to play a role in the development of severe malaria as a consequence of blockage of the blood flow, yet the biological advantage of rosetting for the parasite has not been identified. We have here studied rosetting in 76 patient isolates and propose rosetting to enhance parasite multiplication and to be a likely reason for the existence of rosetting. Further, the results achieved from the comparative growth of clinical isolates demonstrate that optimal *in vitro* conditions to ensure good outgrowth, multiplication, rate of multiple invasions and rosetting include growth in suspension, the addition of human serum and a fixed micro-aerophilic environment. Serum was found necessary for optimal presentation of PfEMP1 at the pRBC surface and maintenance of the adhesive phenotype of rosetting for some parasite strains. Numerous other parameters, including variations of the ones presented here, and the quality of the cryo-preservation and thawing procedures are most likely also crucial factors in the success of establishing clinical isolates *in vitro*. Different aspects of these were not investigated here, instead were all isolates snap frozen in liquid nitrogen in the same manner after collection and thawed according to standard procedures [43].

All isolates studied here were successfully established for *in vitro* growth from frozen aliquots and scored for rosetting at least five times over each developmental cycle for the first three consecutive cycles *in vitro.* Slight differences in the results were observed, as expected, compared to those previously obtained [40] as a consequence of optimized culture conditions. In the previous study rosetting rates were monitored after static growth using the candle jar method [40] while we here studied isolates grown in suspension and a controlled micro-aerophilic environment that we also found to be the preferred conditions for both fresh and established parasites for maintaining their phenotypes. Rosetting-rates were higher and survival greater using the optimized protocol and in the present study parameters such as outgrowth and the number of multiple parasitized RBCs did not vary between the two groups of children which suggests unbiased opportunities for group comparisons. This study further emphasizes the importance of human serum during the cultivation of *P. falciparum* isolates and laboratory strains to guarantee the display of the parasite’s adhesive phenotype and correct expression of PfEMP1. Various parasite lines depend on human immunoglobulins for the formation of rosettes [23], [34], [35], [36], [38], [44]; in addition, rosetting requires complementary serum factors [37]. Studies investigating the rosetting phenomena need to take into consideration that the display of the rosetting phenotype and expression of PfEMP1, and possibly other parasite ligands, can be dependent on the presence of human serum. One possible mechanism is the role of lipid components during translocation of the PfEMP1 molecule from the Maurer’s clefts to the pRBC membrane [31], [32].

The role of PfEMP1, and other potential parasite derived ligands, in the intimate interaction between infected and uninfected RBCs in rosettes may prepare the latter for facilitated merozoite invasion by secreted antigens, by bridging molecules or merely by being in the proximity of the pRBC during schizont rupture. This may allow the merozoite to evade certain, but maybe not all, anti-merozoite immune responses in highly rosetting parasites. The surprising presence in all fresh isolates of high levels of schizont rosettes could suggest that multiple PfEMP1s are erythrocyte adhesive and the acquisition of antibodies towards those PfEMP1s may be involved in the early development of immunity to severe disease in endemic areas. Still, contribution by other parasite ligands in schizont rosetting can not be excluded, considering the presence of several parasite derived proteins at the infected erythrocyte surface prior to and during rupture of the infected cell (reviewed by Cooke et al [45]).

The peripheral parasitemia, the level of rosetting and the rate of multiplication correlated positively to one another for individual isolates and were all found to be higher among isolates from children with severe than from children with uncomplicated malaria. This suggests rosetting to contribute to a higher parasite burden *in vivo*. The comparative advantage a rosetting parasite carries over a non-rosetting one in the ability to invade erythrocytes could have a profound effect on the amplification of the parasite mass in a patient, and thus on the potential to bring about severe malaria. Still, inhibition of rosetting employing anti-PfEMP1 antibodies *in vitro* did not affect invasion here. Thus, other factors, such as merozoite targeted immunity and the high shear force that parasites are exposed to *in vivo*, might be needed in order for the rosetting phenotype to be clearly advantageous for invasion and multiplication. In addition, other traits than rosetting such as the rate of replication, numbers of formed merozoites and the invasion ligand repertoire are also likely to contribute to the superior growth of some but not all parasites.

Taken together, we suggest that rosetting facilitates successful merozoite invasion, which could explain why this parasite phenotype often found associated to severe disease is frequent and selected for in nature. Antibodies towards PfEMP1, the molecule central for the phenotype of rosetting, and immunity to severe malaria develop after only one or a few infections, suggesting development of immunity to PfEMP1 may be important in preventing both the multiplication of the parasite and the microvascular clogging seen in severe malaria. The contribution to parasite growth by highly rosetting isolates *in vivo* could explain the results presented, similar to what have been seen *in vivo* in primates carrying high loads of parasites in their circulation [20]. The difference of advantage of rosetting for invasion in *in vivo* as compared to *in vitro* conditions could depend on more complex mechanisms regulating invasion in the malaria patient as compared to culture conditions. Similarly, contradictory results have been observed before, where our data corroborate previous work [19], [20], but other studies [46] did not find an association between parasite multiplication rates and severity of malaria and could not find greater invasion with rosetting versus non-rosetting parasites [35], maybe due to differences in the *in vitro* culture conditions as compared to *in vivo*.

## Materials and Methods

### Ethics Statement

The study was approved by Karolinska Institute’s Regional Ethical Review Board (permission 03/095) and the Uganda National Council for Science and Technology (permission MV717). Written informed consent was obtained from the parents or guardians of the patients.

### Parasites

Parasites used were long-term propagated parasite strains not selected for rosetting or cytoadhesion (HB3, F32, 3D7AH1, FCR3, R29 and TM284), parasite clones selected for high or low rosetting by micro-manipulation (FCR3S1.2, FCR3S1.6, TM284S2 and 3D7S8.4) and 76 clinical isolates collected in Apac, Uganda 2002 [40], see table S1. The isolates were all randomly selected from unique patients and were shown to be genetically distinct by *msp1*, *msp2*, *glurp* and *csp* genotyping (data not shown). Patients were under the age of five and subdivided into two groups, severe (n = 36) or uncomplicated (n = 40) *falciparum* malaria, using WHO guidelines [47] and the modified Blantyre score [48]. The parasitemia and the number of multiple infected RBCs were estimated from Giemsa-stained thin smears obtained directly from the peripheral blood of the patient [40].

### Evaluation of *in vitro* Growth conditions for Thawed Clinical Isolates

Ten clinical isolates were thawed according to standard procedures and transferred to 5 ml malaria culture medium [43], [49] (tissue culture flasks 25cm^2^, Falcon, nr. 353082) containing 12.5% non-immune human AB^+^ serum and sub-cultivated in fresh O^+^ RBCs during the first cycle *in vitro* (see below for details). Each sub-cultivated isolate were subjected to four different culture conditions (all at 37°C) involving the use of a gas mixture (5% O_2_ and 5% CO_2_ in N_2_), classical candle jar [49], growth in static manner, growth in suspension on an orbital shaker (50 rpm) [50], [51] or combinations thereof. The differently treated cultures were evaluated for outgrowth, the rate of multiple parasitized RBCs, multiplication rates and rosetting rates (see below for details).

### Evaluation of Parasite Outgrowth, Multiple Infected RBCs, Parasite Multiplication Rates and Rosette Formation

All 76 isolates were upon thawing successfully grown until mature trophozoite stages before sub-cultivated at least 1∶10 to a parasitemia in the range 0.1–0.3% in fresh O^+^ RBCs. The same serum batch was used as supplement for all cultures, and an equal number of uncomplicated and severe isolates were cultured in the same batch of O^+^ RBCs (both serum and RBCs were pooled from at least three individual donors to counteract oddities of individual donors and to remove RBC polymorphisms as potential confounders). Rosetting rates were assessed as previously described [43] by staining 10 µl drops of resuspended parasite culture (0.5 - 2.5% parasitemia) with 2µl acridine orange (10µg/ml in PBS). Rosetting rates were counted from at least 100 pRBCs in duplicate in a Nikon Optiphot 2 Phase Fluorescence microscope (Nikon) and pictures were taken using a Hamamatsu Color Chilled 3CCD Camera (Hamamatsu Photonics). A trophozoite rosette was defined as two or more uninfected RBCs bound to one or two trophozoite-infected RBCs and a schizont rosette as two or more uninfected RBCs bound to one or two schizont-infected RBCs. The parasites were analyzed five times during intra-RBC progression (0–10, 10–20, 20–30, 30–38 and 38–48 hours post invasion) for the three first parasite generations. The rosetting-rates presented for each time-point are the averages of the first three consecutive parasite generations post thawing. Thin smears stained with Giemsa were prepared when cultures were in ring stages. Parasitemia was determined in at least 5,000 RBCs for the original culture. The same was performed on both cultures after sub-cultivation and after invasion in the next consecutive parasite generation in order to compute multiplication rates. Further, the number of viable versus pyknotic parasites and the number of multiple infected RBCs was assessed. We have previously reported the trophozoite rosetting rates for the samples studied here after cultivation under static conditions using the candle-jar method in Kampala (Uganda) [40]. The parasites studied herein are from cryo-preserved stocks of the same samples propagated on an orbital shaker (50 rev/min) with the use of a fixed gas composition (5% O2, 5% CO2 and 90% N2) in Stockholm (Sweden).

For the evaluation of the importance of human serum for rosetting and correct PfEMP1 surface expression, parasites were grown in parallel in malaria complete medium containing 10% human serum, or 0.5% Albumax II respectively.

### Analysis of Surface Expression of PfEMP1 by Flow Cytometry

Trophozoite-infected RBCs selected at seven different time-points (18, 22, 26, 30, 34, 38 and 42 h p.i.) were incubated in a final concentration of 50 ug/ml with a strain-specific mAb (α-NTS-DBL1α_var2_ [39]) diluted in PBS/FCS (2%) for 30 min at RT. After incubation with the primary antibody, three washes with PBS/FCS was performed followed by 30 min incubation with anti-mouse IgG second antibody ALEXA488 (Molecular Probes, dilution 1∶100) at RT. For nuclear staining ethidium bromide was added at final concentration of 2.5µg/ml for 5 min at RT. The pRBCs were washed three times and resuspended in PBS/FCS. The cell acquisition was done using flow cytometry (FACSCalibur, BD Bioscience, http://www.bd.com) where 5000 pRBCs were counted. The analysis was performed using the software FlowJo. Binding of non-immune human Ig was visualized as above using an anti-human Ig FITC-coupled antibody (DAKO, F0200, dilution 1∶100). As a negative control a monoclonal antibody generated in mouse against a non-related bacterial protein has been used in all experiments.

### Invasion Assays

PRBCs of the parasite strain FCR3S1.2 at trophozoite stage were adjusted to a parasitemia of 0.5% and a hematocrit of 2.5%, mixed with the purified IgG fraction of an α-NTS-DBL1α_var2_ goat serum, a α-NTS-DBL1α_var2_ mAb (1, 0.5 and 0.25 mg/ml, in duplicates) and cultivated in 96-well plates until reinvasion of merozoites was completed. The applied antibodies were α-NTS-DBL1α_var2_ mouse mAb or purified polyclonal goat IgG generated against and specific for the DBL1α-domain of ITvar60 expressed by FCR3S1.2 pRBCs [39].

Parasitemia was measured at ring stage (around 15 h p.i.) and assays were performed under shaking versus non shaking conditions under a fixed gas composition. In addition, goat IgG was pre-absorbed by incubation with uninfected RBCs for 30 min at RB prior to the invasion experiments. As a negative control, normal goat IgG or mouse IgG was used in the same concentrations.

PRBCs were stained with Acridine Orange and counted by flow cytometry (50,000 events). Experiments were repeated three times and the effect was determined by relating the observed parasitemia in the presence of PfEMP1_var2_-specific antibodies to invasion in the presence of corresponding non-immune immunoglobulins.

### Invasion into Enzyme Treated Erythrocytes

Human O+ erythrocytes were washed with RPMI 1640 (Gibco, Invitrogen), and subsequently incubated with neuraminidase (62.5 uM/ml; Sigma), trypsin (1mg/ml; Sigma) and chymotrypsin (1 mg/ml; Sigma) for 45 minutes at 37 ^0^C with periodic shaking. Controls were only treated with RPMI 1640. After incubation, enzyme treated cells were washed once with RPMI 1640 containing 20% human serum and twice with RPMI 1640 containing 10% human serum to inhibit enzyme activity [52].

Late-pigmented trophozoites to schizont stage were enriched using magnetic bead column separation (Miltenyi Biotec, Germany). Invasion assay was performed in 96 U-bottom culture plates with total of 50 µl of parasite suspension at 0.5–1.0% parasitemia and enzyme treated erythrocytes. All samples were tested in triplicate. Plates were incubated in a gassed box and incubated for 48 hours at 37°C. Parasitemia was estimated using hydroethidine (10 ug/ml; Sigma) in flow cytometry (FACS Scan; BD) after 48 hours. Inhibition by enzyme treatment was determined as [1 − (proportion of enzyme-treated cells invaded/proportion of untreated cells invaded)] × 100. Results presented are in comparison with control treated cells [52].

### Statistical Analysis

SigmaStat 3.1 (Systat Software, Erkrath, Germany) was used for statistical analyses. Group comparisons (severe vs. uncomplicated) for parasite multiplication rate and rosetting at mid-trophozoite stage were analyzed using the non-parametric Mann-Whitney rank sum test due to non-normality of the data, whereas all other group comparisons were conducted using Student’s t-test. Correlations between multiplication rates and rosetting rates as well as multiplication rates and original peripheral parasitemia were assessed using Spearman rank correlations. The four culture conditions were evaluated and compared using one way ANOVA with pairwise multiple comparisons using the Tukey for all generations. The temporal differences in rosetting (trophozoites vs schizonts) among the clinical isolates were analyzed by Kruskal-Wallis ANOVA on ranks with pairwise multiple comparison procedures using the Tukey test. A priori assumption of statistical significance was in all cases determined to *p*<0.05.

### Transmission Electron Microscopy

Transmission electron microscopy (TEM) was used to visualize rosettes at both trophozoite and schizont stages. RBCs and pRBCs of the clinical isolate UAS29 were fixed in 2% glutaraldehyde and 0.5% paraformaldehyde in 0.1 M sodiumcacodylate buffer containing 0.1 M sucrose (pH 7.4) at room temperature for 30 min. Fixed cells were rinsed in 0.15 M sodiumcacodylate buffer (pH 7.4), pelleted by centrifugation, post fixed in 2% osmium tetroxide buffer (pH 7.4) at 4°C for 2 hours, dehydrated in ethanol and acetone before embedded in LX-112 (Ladd Research Industries, Burlington, VT, USA). Sections were contrasted with uranyl acetate followed by lead citrate and examined in Leo 906 transmission electron microscope at 80 kV. Digital images were taken using a Morada digital camera (Olympus Soft Imaging System, Münster, Germany) [53].

### Time-lapse Capture of Rupture and Invasion from Rosettes

The time-lapse movie was made using an automated confocal microscope, a custom modified LCI system (Perkin-Elmer, Upplands Väsby, Sweden). The system includes a motorized Axiovert fluorescence microscope 200 M (Zeiss, Göttingen, Germany), a CSU10 Nipkow spinning disc (Yokogawa, Japan), a motorized XY-table (Märzhauser), an ORCA ER cold CCD camera; detector array 1344 x 1024 px (Hamamatsu, Hamamatsu City, Japan), a 3-line Argon-Krypton Laser; 488 nm, 568 nm, 647 nm (Melles Griot, Stockholm, Sweden) and halogen illumination for bright field and phase contrast mode. A 63X/NA 1.25 Oil Ph3 Plan – Neofluor ∞/0.17, Zeiss objective was used. The images were captured using the movie automation FiveColorMovie with autofocus function, developed by authors (EF+LS) in the visual programming language environment of Openlab automator. For each time-point, 4 phase-contrast images (350 ms exposure/image) were captured through a 1µm thick z-depth (0.3 µm/step). The interval between time-points was 30 seconds.

## Supporting Information

Figure S1 **Pairwise multiple comparisons of parasite growth and rosetting for the four evaluated culture conditions.** Statistical analyses of the data presented in Figure 1, describing the differences in parasite outgrowth, multiple invaded RBCs, parasite multiplication and rosetting for all parasite generations (G0–G20) analyzed. The different conditions were 1) growth in suspension (50 rev/min) with fixed gas composition (5% O2 and 5% CO2 in N2) (•), 2) the same but with first generation grown under static conditions (○), 3) static growth with fixed gas (▾) and 4) static growth with candle jar technique (Δ). All statistically significant pairwise multiple comparisons are shaded in light green (p<0.05), and non-significant data in orange. Quadrants lacking numbers indicate too small differences in mean values among the groups and therefore failed test due to low power.
(PDF)

Figure S2 **Time-lapse capture of schizont rupture from a rosette under static growth.** Timing of schizont rupture in rosetting FCR3S1.2. Four phase-contrast images were captured through a 1 µm thick z-depth using a Nipkow spinning disc confocal microscope with 30 second intervals between time-points, visualizing the invasion of bound erythrocytes from schizont rosettes upon rupture. In total 5.5 minutes of real-time capture is represented, with timing indicated in the bottom-right of each panel. The time-laps capture can also be viewed as continuous in Movie S1. Panel 1 (0–1 min): A schizont pRBC (white arrow) ruptures after 1 min while still attached to five RBCs, with a concomitant egress of merozoites. A few free merozoites are visual (red arrows). Panel 2 (1.5–2.5 min): Four of the bound RBCs are immediately invaded by merozoites (green arrows), while free merozoites can still be seen (red arrows). Panel 3 (3–4 min): The cells from the original rosette still attach to the remnants of the ruptured pRBC and each other and the fifth RBC gets invaded by merozoite (green arrow). Panel 4 (4.5–5.5 min): All of the RBCs from the original rosette have been turned into pRBCs (white arrows) and still surround and attach to the remnant pRBC ghost and each other, albeit the interactions are weakening.
(TIF)

Figure S3 **Correlation between rosetting and invasion in the laboratory strain FCR3S1.2.** Invasion inhibition using rosette-disruptive antibodies against the PfEMP1 variant displayed by FCR3S1.2. Goat IgG αNTS-DBL1α, non-absorbed or pre-absorbed on RBCs or monoclonal αNTS-DBL1α antibodies were added to early trophozoite stage pRBCs at concentrations of 1, 0.5 and 0.25 mg/ml, parasites were grown with gas under shaking conditions, allowed to invade and parasitemia was measured thereafter. Non-related antibodies from the same species were used as controls. The level of invasion was comparable in the presence or absence of antibodies blocking invasion and no correlation between the level of rosetting and invasion could be observed for this long term propagated laboratory strain. Graph shows median of three experiments with range.
(PDF)

Table S1 **Summary of patient and** ***P. falciparum*** **isolate data.**
(DOC)

Movie S1 **Time-lapse capture of schizont rupture and merozoite invasion from rosettes.** Timing of schizont rupture and merozoite invasion in rosetting FCR3S1.2. Four phase-contrast images were captured through a 1 µm thick z-depth using a Nipkow spinning disc confocal microscope with 30 second intervals between time-points, visualizing the invasion of bound erythrocytes from schizont rosettes upon rupture. In total 7.5 min of real-time capture is represented, with timing indicated in the bottom-right of the screen. Events shown in this movie are described in detail in figure legend for Figure S2.
(MOV)
