# Supplementary figures and images for: Improved In Vitro Culture of Plasmodium falciparum Permits Establishment of Clinical Isolates with Preserved Multiplication, Invasion and Rosetting Phenotypes
Source: PLoS One. 2013 Jul 22;8(7):e69781. doi: 10.1371/journal.pone.0069781 (PMC3718792; doi:10.1371/journal.pone.0069781)

Supplementary Figure 1

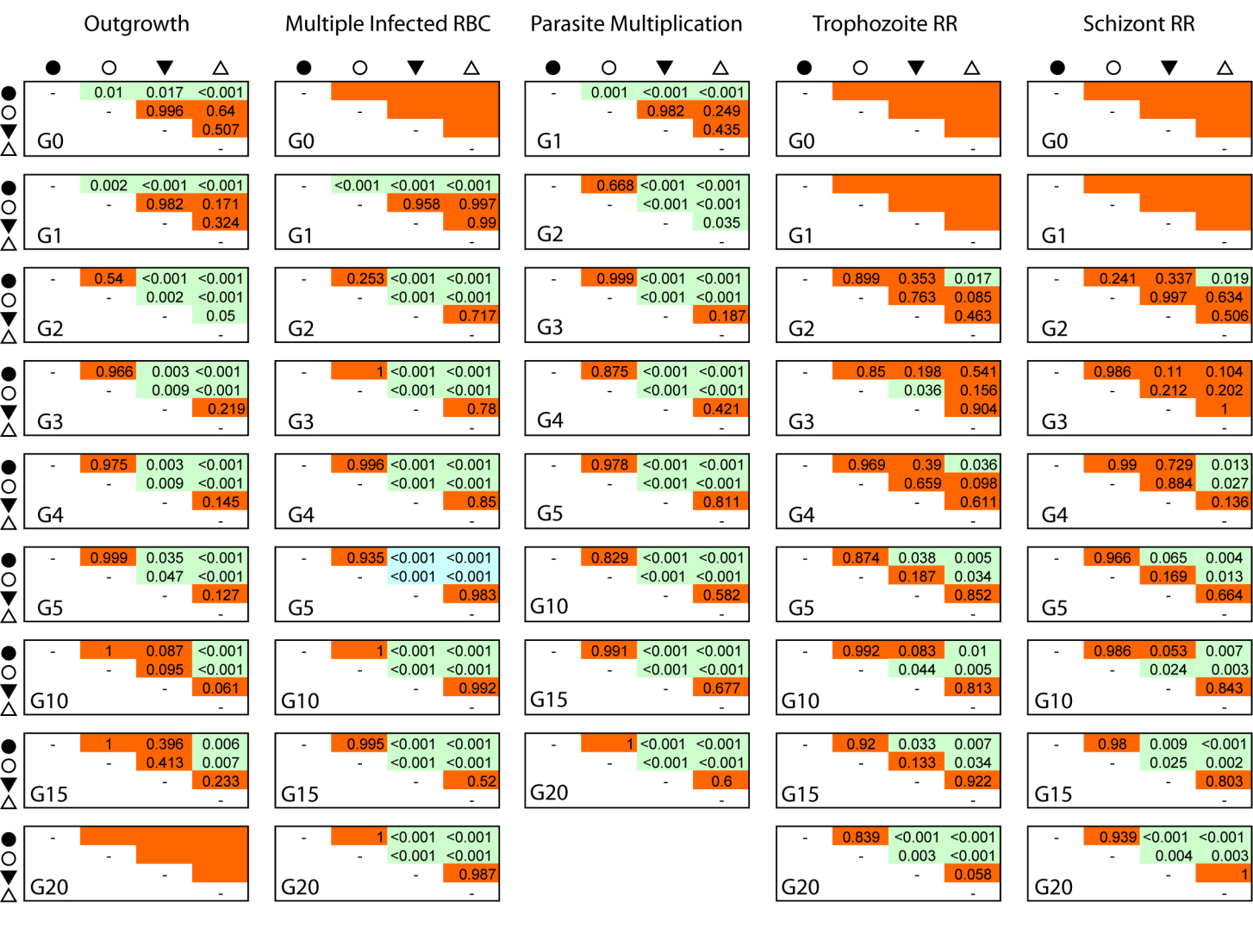

Supplement: Figure S1 — Pairwise multiple comparisons of parasite growth and rosetting for the four evaluated culture conditions. Statistical analyses of the data presented in Figure 1, describing the differences in parasite outgrowth, multiple invaded RBCs, parasite multiplication and rosetting for all parasite generations (G0–G20) analyzed. The different conditions were 1) growth in suspension (50 rev/min) with fixed gas composition (5% O2 and 5% CO2 in N2) (•), 2) the same but with first generation grown under static conditions (○), 3) static growth with fixed gas (▾) and 4) static growth with candle jar technique (Δ). All statistically significant pairwise multiple comparisons are shaded in light green (p<0.05), and non-significant data in orange. Quadrants lacking numbers indicate too small differences in mean values among the groups and therefore failed test due to low power. (PDF) [file pone.0069781.s001.pdf]

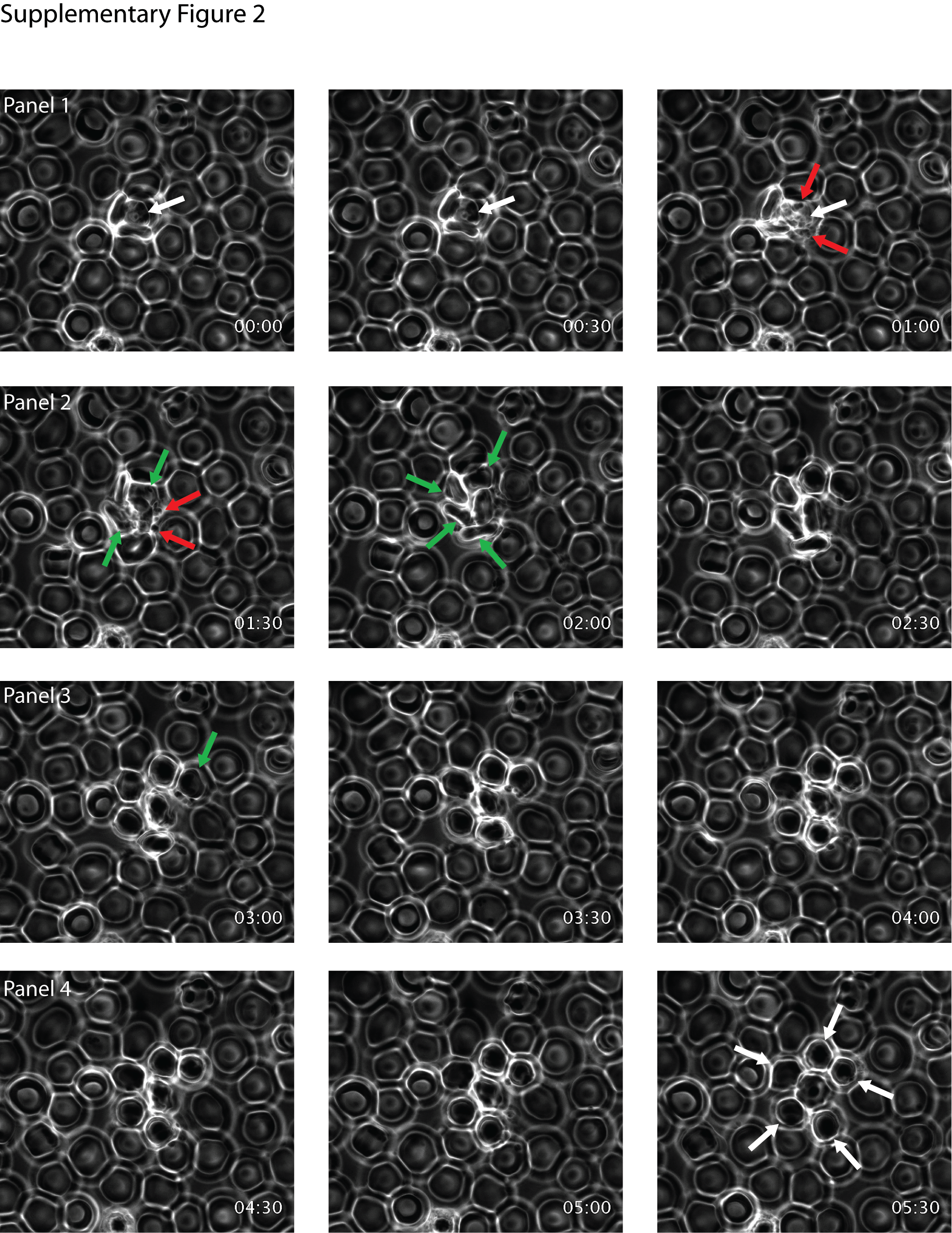

Supplement: Figure S2 — Time-lapse capture of schizont rupture from a rosette under static growth. Timing of schizont rupture in rosetting FCR3S1.2. Four phase-contrast images were captured through a 1 µm thick z-depth using a Nipkow spinning disc confocal microscope with 30 second intervals between time-points, visualizing the invasion of bound erythrocytes from schizont rosettes upon rupture. In total 5.5 minutes of real-time capture is represented, with timing indicated in the bottom-right of each panel. The time-laps capture can also be viewed as continuous in Movie S1. Panel 1 (0–1 min): A schizont pRBC (white arrow) ruptures after 1 min while still attached to five RBCs, with a concomitant egress of merozoites. A few free merozoites are visual (red arrows). Panel 2 (1.5–2.5 min): Four of the bound RBCs are immediately invaded by merozoites (green arrows), while free merozoites can still be seen (red arrows). Panel 3 (3–4 min): The cells from the original rosette still attach to the remnants of the ruptured pRBC and each other and the fifth RBC gets invaded by merozoite (green arrow). Panel 4 (4.5–5.5 min): All of the RBCs from the original rosette have been turned into pRBCs (white arrows) and still surround and attach to the remnant pRBC ghost and each other, albeit the interactions are weakening. (TIF) [file pone.0069781.s002.tif]

Supplementary Figure 3

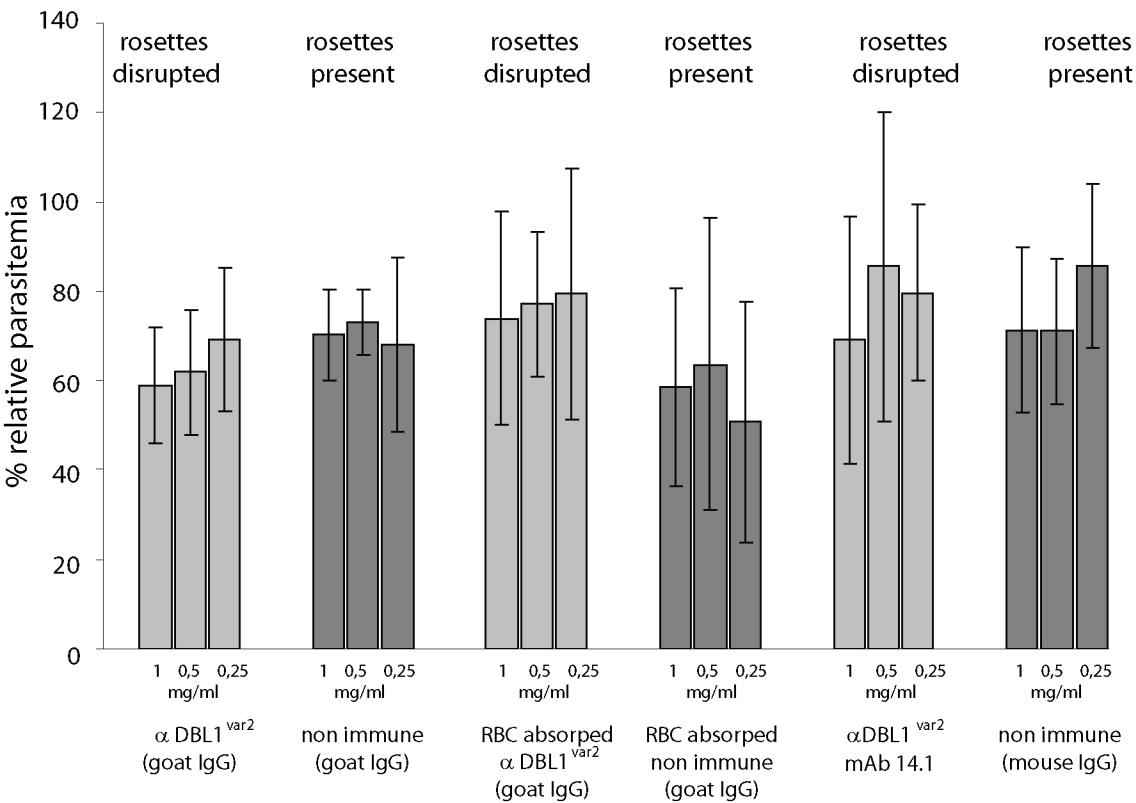

Supplement: Figure S3 — Correlation between rosetting and invasion in the laboratory strain FCR3S1.2. Invasion inhibition using rosette-disruptive antibodies against the PfEMP1 variant displayed by FCR3S1.2. Goat IgG αNTS-DBL1α, non-absorbed or pre-absorbed on RBCs or monoclonal αNTS-DBL1α antibodies were added to early trophozoite stage pRBCs at concentrations of 1, 0.5 and 0.25 mg/ml, parasites were grown with gas under shaking conditions, allowed to invade and parasitemia was measured thereafter. Non-related antibodies from the same species were used as controls. The level of invasion was comparable in the presence or absence of antibodies blocking invasion and no correlation between the level of rosetting and invasion could be observed for this long term propagated laboratory strain. Graph shows median of three experiments with range. (PDF) [file pone.0069781.s003.pdf]
