# Supplementary material for: Improved In Vitro Culture of Plasmodium falciparum Permits Establishment of Clinical Isolates with Preserved Multiplication, Invasion and Rosetting Phenotypes
Source: PLoS One. 2013 Jul 22;8(7):e69781. doi: 10.1371/journal.pone.0069781 (PMC3718792; doi:10.1371/journal.pone.0069781)
Supplement: Table S1 — Summary of patient and P. falciparum isolate data. (DOC) [file pone.0069781.s004.doc]

**Supplementary table 1**

Summary of patient and *P. falciparum* isolate data

**severe isolates**

| **Isolate** | **Collection date** | **Sex** | **Age**  **(months)** | **Initial parasitemia (%)** | **Trophozoite rosetting rate*** **(%)** | **Schizont rosetting rate**^†^ **(%)** | **Disease state group^φ^** |
| --- | --- | --- | --- | --- | --- | --- | --- |
|  |  |  |  |  |  |  |  |
| UAS06 | 11/4/02 | M | N/A | 9.1 | 9 | 38 | Severe malaria NUD |
| UAS07 | 11/4/02 | F | 11 | 9 | 45 | 61 | Respiratory distress |
| UAS08 | 11/4/02 | F | 16 | 14.8 | 37 | 53 | Respiratory distress |
| UAS09 | 11/4/02 | M | 17 | 15 | 15 | 42 | Respiratory distress |
| UAS11 | 11/7/02 | F | 4 | 46 | 31 | 50 | Severe malaria NUD |
| UAS13 | 11/7/02 | M | 12 | 5 | 56 | 65 | Respiratory distress |
| UAS14 | 11/7/02 | F | 15 | 10 | 50 | 79 | Severe malaria NUD |
| UAS19 | 11/14/02 | F | 14 | 1.1 | 44 | 54 | Respiratory distress |
| UAS20 | 11/14/02 | M | 6 | 1.9 | 3 | 30 | Severe malaria NUD |
| UAS21 | 11/14/02 | M | 8 | 9 | 53 | 61 | Respiratory distress |
| UAS22 | 11/18/02 | M | 17 | 21.8 | 33 | 57 | Respiratory distress |
| UAS23 | 11/18/02 | M | 20 | 4 | 9 | 31 | Cerebral malaria |
| UAS24 | 11/18/02 | F | 15 | 5 | 57 | 64 | Respiratory distress |
| UAS25 | 11/18/02 | M | 8 | 5.2 | 7 | 30 | Respiratory distress |
| UAS26 | 11/18/02 | F | 6 3. | 1 | 12 | 40 | Respiratory distress |
| UAS28 | 11/21/02 | F | 5 | 6.1 | 10 | 31 | Respiratory distress |
| UAS29 | 11/21/02 | F | 12 | 3.9 | 85 | 88 | Respiratory distress |
| UAS30 | 11/21/02 | M | 19 | 2 | 5 | 42 | Severe malaria NUD |
| UAS31 | 11/21/02 | M | 30 | 7.6 | 80 | 81 | Severe malaria NUD |
| UAS32 | 11/25/02 | M | 9 | 6.2 | 6 | 37 | Cerebral malaria |
| UAS33 | 11/25/02 | F | 14 | 2.8 | 6 | 37 | Respiratory distress |
| UAS34 | 11/25/02 | F | 8 | 10 | 28 | 57 | Respiratory distress |
| UAS35 | 11/25/02 | M | 30 | 8.3 | 42 | 56 | Respiratory distress |
| UAS36 | 11/27/02 | F | 5 | 9.3 | 30 | 65 | Respiratory distress |
| UAS37 | 11/27/02 | F | 14 | 5 | 28 | 43 | Respiratory distress |
| UAS38 | 11/29/02 | M | 30 | 9.5 | 2 | 30 | Severe malaria NUD |
| UAS39 | 11/29/02 | M | 6 | 9.3 | 32 | 53 | Respiratory distress |
| UAS40 | 11/29/02 | M | 9 | 5.5 | 5 | 32 | Respiratory distress |
| UAS41 | 12/2/02 | F | 11 | 7.9 | 49 | 59 | Circulatory collapse |
| UAS42 | 12/2/02 | M | 9 | 2.2 | 66 | 69 | Respiratory distress |
| UAS43 | 12/2/02 | F | 14 | 5.6 | 43 | 52 | Respiratory distress |
| UAS44 | 12/2/02 | M | 5 | 4.5 | 7 | 40 | Respiratory distress |
| UAS46 | 12/2/02 | M | 9 | 1.8 | 46 | 71 | Respiratory distress |
| UAS47 | 12/2/02 | F | 7 | 1.3 | 37 | 48 | Severe anemia |
| UAS48 | 12/4/02 | M | 15 | 7.3 | 56 | 75 | Respiratory distress |
| UAS49 | 12/4/02 | M | 9 | 5.7 | 21 | 46 | Respiratory distress |
| **n=36** |  |  |  |  |  |  |  |
|  |  |  | 12.5 | 7.8 | 31.7 | 51.9 |  |

**uncomplicated isolates**

| **Isolate** | **Collection date** | **Sex** | **Age**  **(months)** | **Initial parasitemia (%)** | **Trophozoite rosetting rate***  **(%)** | **Schizont rosetting rate**^†^ **(%)** | **Disease state group^φ^** |
| --- | --- | --- | --- | --- | --- | --- | --- |
| **UAM05** | 11/4/02 | F | 12 | 1 | 9 | 34 | Uncomplicated malaria |
| **UAM06** | 11/4/02 | M | 36 | 2 | 6 | 31 | Uncomplicated malaria |
| **UAM07** | 11/4/02 | M | 36 | 2 | 7 | 43 | Uncomplicated malaria |
| **UAM08** | 11/7/02 | F | 36 | 2 | 49 | 65 | Uncomplicated malaria |
| **UAM09** | 11/7/02 | F | 4 | 2.4 | 31 | 53 | Uncomplicated malaria |
| **UAM11** | 11/7/02 | M | 14 | 2.8 | 3 | 33 | Uncomplicated malaria |
| **UAM13** | 11/11/02 | F | 5 | 1 | 10 | 32 | Uncomplicated malaria |
| **UAM14** | 11/14/02 | F | 35 | 1.7 | 4 | 28 | Uncomplicated malaria |
| **UAM15** | 11/14/02 | M | 24 | 1.4 | 11 | 42 | Uncomplicated malaria |
| **UAM16** | 11/14/02 | F | 9 | 1.6 | 38 | 68 | Uncomplicated malaria |
| **UAM17** | 11/18/02 | M | 9 | 1 | 19 | 35 | Uncomplicated malaria |
| **UAM18** | 11/18/02 | M | 27 | 2 | 13 | 46 | Uncomplicated malaria |
| **UAM19** | 11/18/02 | F | 24 | 1 | 8 | 41 | Uncomplicated malaria |
| **UAM20** | 11/21/02 | M | 27 | 1 | 3 | 35 | Uncomplicated malaria |
| **UAM21** | 11/21/02 | M | 6 | 3.2 | 25 | 51 | Uncomplicated malaria |
| **UAM23** | 11/21/02 | M | 36 | 2 | 3 | 30 | Uncomplicated malaria |
| **UAM24** | 11/25/02 | F | 5 | 6.7 | 11 | 43 | Uncomplicated malaria |
| **UAM25** | 11/25/02 | F | 11 | 4.2 | 11 | 34 | Uncomplicated malaria |
| **UAM28** | 11/25/02 | M | 24 | 2.3 | 7 | 41 | Uncomplicated malaria |
| **UAM29** | 11/27/02 | F | 12 | 2.5 | 68 | 71 | Uncomplicated malaria |
| **UAM31** | 11/27/02 | F | 9 | 1.9 | 1 | 27 | Uncomplicated malaria |
| **UAM32** | 11/27/02 | F | 20 | 1 | 0 | 44 | Uncomplicated malaria |
| **UAM33** | 11/29/02 | F | 7 | 3.8 | 9 | 34 | Uncomplicated malaria |
| **UAM34** | 11/29/02 | M | 7 | 2 | 57 | 74 | Uncomplicated malaria |
| **UAM35** | 11/29/02 | F | 10 | 1.2 | 2 | 37 | Uncomplicated malaria |
| **UAM36** | 11/29/02 | M | 12 | 4.8 | 7 | 39 | Uncomplicated malaria |
| **UAM37** | 11/29/02 | M | 48 | 0.8 | 3 | 36 | Uncomplicated malaria |
| **UAM38** | 11/29/02 | M | 8 | 1.8 | 7 | 29 | Uncomplicated malaria |
| **UAM39** | 11/29/02 | F | 7 | 1.5 | 13 | 36 | Uncomplicated malaria |
| **UAM40** | 12/2/02 | F | 24 | 5.1 | 8 | 31 | Uncomplicated malaria |
| **UAM42** | 12/2/02 | M | 9 | 5.9 | 22 | 45 | Uncomplicated malaria |
| **UAM43** | 12/2/02 | M | 8 | 4.1 | 7 | 31 | Uncomplicated malaria |
| **UAM44** | 12/4/02 | M | 6 | 2.3 | 5 | 32 | Uncomplicated malaria |
| **UAM45** | 12/4/02 | F | 29 | 1.8 | 27 | 49 | Uncomplicated malaria |
| **UAM47** | 12/4/02 | F | 12 | 1.3 | 33 | 56 | Uncomplicated malaria |
| **UAM48** | 12/4/02 | M | 19 | 2.8 | 0 | 34 | Uncomplicated malaria |
| **UAM49** | 12/4/02 | F | 4 | 10 | 29 | 47 | Uncomplicated malaria |
| **UAM50** | 12/4/02 | F | 20 | 3.6 | 4 | 32 | Uncomplicated malaria |
| **UAM51** | 12/4/02 | F | 6 | 1.6 | 36 | 48 | Uncomplicated malaria |
| **UAM52** | 12/4/02 | M | 6 | 2 | 9 | 26 | Uncomplicated malaria |
| **n=40** |  |  |  |  |  |  |  |
| **Mean** |  |  | 14.2 | 2.6 | 15.4 | 41.1 |  |

* Rate of rosetting at 20-30 hours post invasion. Average from the first three consecutive generations post thawing.

† Rate of rosetting at 38-48 hours post invasion. Average from the first three consecutive generations post thawing.

φ Severe malaria NUD: Severe malaria non ultra descriptus (included patients with convulsions, prostration, hyperparasitemia, and hyperpyrexia)
